# Exploring nurses’ experiences of recommended patient care: a descriptive phenomenological study

**DOI:** 10.1186/s12912-024-01736-z

**Published:** 2024-01-22

**Authors:** Azam Faraji, Amir Jalali, Alireza Khatony, Rostam Jalali

**Affiliations:** 1 Nursing Department, Kermanshah University of Medical Sciences, Kermanshah, Iran; 2 Psychiatric Nursing Department, School of Nursing and Midwifery, Kermanshah University of Medical Sciences, Kermanshah, Iran; 3 Social Development and Health Promotion Research Center, Kermanshah University of Medical Sciences, Kermanshah, Iran

**Keywords:** Recommended patient, Nurses, Very important person, Qualitative research, Lived experiences

## Abstract

**Background:**

Caring for recommended patients creates work and emotional challenges for nurses. Nurses are obligated to provide care regardless of the patient’s situation. Therefore, knowing the experiences of nurses in dealing with recommended patients in order to provide quality and effective care can be the basis for increasing patient satisfaction. The present study was conducted aimed to explain nurses’ experiences of caring for recommended patients.

**Methods:**

This was a qualitative study with descriptive phenomenological approach. Participants were 12 nurses working in different wards of hospitals affiliated to Kermanshah University of Medical Sciences, selected by purposive sampling method with maximum diversity. The data collected using semi-structured interviews in face-to-face and audio-recorded methods. MAXQDA 2020 software was used for data management. The analysis of the data was done using the Colaizzi’s 7-step method. In order to verify the trustworthiness of the data, Lincoln and Guba criteria were used.

**Results:**

After continuous data analysis, 110 initial codes were extracted. These codes emerged in 18 sub-themes and 6 main themes including: catastrophe, be in decline, be in progress, discrimination, work overload, and poor prognosis.

**Conclusions:**

The results showed information about the presence of recommended patients in the hospital, which can have consequences for patients and nurses. Therefore, it is advised that nurses provide standard care and avoid any kind of discrimination against all patients regardless of whether the patient is recommended or not.

## Introduction

Caring for very important persons (VIPs), including celebrities and royalty, is associated with medical, organizational and administrative challenges and is often collectively known as the “VIP syndrome”. A situation that often forces the healthcare team to change the rules by which they usually provide healthcare [1]. VIP syndrome has been coined to describe a cycle of patient demands that lead to incorrect clinical judgment in an attempt to meet unrealistic expectations, leading to detrimental outcomes. Expecting specific treatment and emphasizing more tests and interventions often leads to harmful implications and the need for more and often unnecessary medical interventions perpetuates this dangerous cycle [2]. Since the visitors are often VIPs and even superiors of the treating physician, their opinions cannot be ignored immediately [3]. A VIP patient is someone who causes the physician to feel fear (e.g., experience anxiety or tachycardia), by definition, a friend, family member, or physician colleague may be a VIP patient [4]. Recommended patient is a special person who is recommended to the treatment staff because of kinship, social status, and reputation, with the aim of receiving more detailed care [5].

Nurses are the largest group of employees providing health services [6] and the main basis of the process of improving the quality of care services. Accordingly, their performance plays a decisive role in the progress of the organization’s goals [7]. Since patients have the most contact with nurses, some experts attribute the acceptability of health services exclusively to nurses, and the prominent role of other treatment groups is often ignored [8]. All patients have the right to receive quality services and all nurses are responsible for facilitating this issue [9]. In addition, nurses must be legally and ethically accountable for the quality of care provided [10]. The quality of nursing care is the nurse’s response to the physical, psychological, emotional, social, and spiritual needs of patients so that patients can return to their healthy and normal lives while being satisfied [11]. Social justice in the health care system refers to the provision of equal health care services to all people, regardless of their personal characteristics [12]. Previous studies in this field have shown that caring for VIP patients is challenging and stressful, and more attention and access to resources are easier for these patients, and negative consequences such as harming the patient, interruption in care, and increased workload follow Nurses [13–16]. However, according to the approach of this research in relation to recommended patients (not VIP), no study was found in this field.

According to the American Association of Critical Care Nurses (AACN), social justice is a fair treatment regardless of economic status, race, ethnicity, age, citizenship, disability, or sexual orientation [13]. Therefore, nurses are obliged to provide care to clients regardless of their economic status, race, ethnicity, age, citizenship, disability, or sexual orientation or personality characteristics. Nurses sometimes have to take care of recommended patients. In such cases, the type and time of care and the demand for providing more care are determined by the patients and adapted to their personal plan. Usually, the recommended patient care leads to a departure from the usual care procedure and ultimately causes fear in the care team. In previous studies, it has been shown that phenomenology has been used to examine experiences related to specific cases such as caring for Covid-19 patients, caring for critical patients, and end-of-life care [17–19]. Phenomenology focuses on lived experiences that are perceived or interpreted by participants. In addition, exploring the experiences of others can reveal insights not previously available, so it is considered a useful method for the purpose of this study. This method provides an in-depth understanding of how nurses care for recommended patients. According to the searches, no qualitative research was found regarding the experiences of nurses in the care of recommended patients. In addition, since the understanding and insight of nurses regarding experience of caring these patients can help in planning for similar patients, Therefore, the present study was conducted aimed to exploring the meaning and nature of nurses’ experiences of recommended patients care.

## Methods

### Study design

A qualitative study approach with a descriptive phenomenological study was conducted in order to explore the experiences of nurses in caring for recommended patients in 2023. Qualitative research is based on facts and people’s views and is done to achieve a rich understanding of a phenomenon as it exists [20]. Phenomenology is a qualitative approach to finding meaning and essence in people’s experiences to facilitate understanding [17].

### Participants

The participants were 12 nurses working in different wards of hospitals affiliated to Kermanshah University of Medical Sciences (KUMS) Kermanshah- West of Iran. Criteria for entering the research were having at least 3 years of clinical experience, having care experience for recommended patient, and expressing consent to participate in the study.

### Data collection

In this study, the samples were selected by purposive sampling method. In this study, according to the nature of qualitative studies [21], samples rich in information were selected to enable a deep investigation of the phenomenon. Interviews continued until reaching data saturation, which was the basis for deciding to stop sampling. This means that by repeating the interviews, no new content was obtained and the data was repetitive [20]. The sampling process lasted about 6 months from February to July 2023. Semi-structured, in-depth interviews were used to collect data. The interviews were conducted in person and face to face. All the interviews were conducted in the place agreed by the researcher and the participant. A comfortable atmosphere was created during the interview by starting everyday conversations. The interview template was developed specifically for this study and the interviews were conducted according to this guide. Interviews began with an open-ended question: “Can you tell me about your experience of caring for recommended patients,” so that participants could describe their experiences in detail and comfortably. Exploratory questions such as why? how? and please explain further, were used to clarify concepts. The interviews lasted from 30 to 60 min depending on the ability of the participants. At the end of each interview, the interviewer mentioned to the participant that a phone call may be needed to discuss the study findings and to ensure that the findings reflect the participants’ experiences. All interviews were conducted by the first author, a woman with a master’s degree in nursing with 12 years of clinical training experience and expertise in qualitative research. All the interviews recorded at the same time. The content of the interviews immediately after each interview was verbatim transcribed and typed. Also, the interviews were carefully listened to by the researchers, the typed text was reviewed several times, then it was coded and finally the concepts were extracted. Data collection and analysis were performed simultaneously.

### Data analysis

MAXQDA 2020 software was used for data management. The data in this study were analyzed with the Colaizzi’s 7-step method, which includes: 1- familiarization with the data, 2- extracting significant statements, 3- formulating meanings, 4- organizing the themes 5- providing a comprehensive description of the phenomenon, 6- identifying the fundamental structure of the phenomenon, and 7- returning to the participants [22].

### Trustworthiness

In this study, to ensure the trustworthiness of the data, Lincoln and Guba evaluative criteria including Credibility, Dependability, Conformability, and Transferability were used [23]. To enhance the credibility the researcher focused on long-term interaction and continuous observation, the researcher devoted a lot of time to collect data, the interview text and the extracted codes were presented to the participants, and they commented on its accuracy and in case of encountering ambiguous points in the data analysis, she conducted a telephone interview to clarify this ambiguity. In order to enhance credibility, the researcher used a qualitative expert colleague to conclude the truth and involved the participants in the study by conducting in-depth interviews with them and also providing the necessary feedback to discover different aspects of the truth. To ensure the dependability of the findings, the researchers used the guidance and supervision of experts. In order to determine the conformability, the researchers tried to avoid confirmation bias, that is, not to support their hypothesis in the interpretation of the data and not removing any results that contradict their opinion. Also, in order to increase the transferability of the findings, sampling with maximum diversity in terms of age, both sexes and at different educational levels was used.

### Ethical considerations

This study received ethics approval from the Research Ethics Committee of Kermanshah University of Medical Sciences (No.IR.KUMS.REC.1401.532). Participants were informed about the purpose of the study, how to report the study results, and audio recording in interviews. After explaining the objectives, written informed consent was obtained from the nurses to participate in the study. In addition, subjects were informed that they were allowed to withdraw from the study at any time without any problems. The place and time of the interview were determined by the participants. Confidentiality of information and maintaining the right to withdraw was meted in all stages of the research. All procedures were performed in accordance with the Declaration of Helsinki guidelines.

## Results

In this study, 58% of the 12 participants were women and 42% were men. More details about the demographic characteristics of the participants are provided in Table 1.

**Table 1:** Demographic characteristics of the participants (*N* = 12)

Participant	Gender	Marital status	Age (years)	Workplace ward	Educational level	Work experience (years)
P1	Female	Married	32	Emergency	BSc	8
P2	Female	single	38	Emergency	MSc	9
P3	Male	Married	40	ICU	BSc	9
P4	Male	single	28	CCU	BSc	4
P5	Female	Married	34	ICU	MSc	8
P6	Female	single	42	ICU	MSc	13
P7	Female	Married	39	Neurosurgery	MSc	13
P8	Male	single	30	ICU	MSc	4
P9	Female	Married	36	CCU	BSc	10
P10	Male	Married	41	Emergency	PhD	12
P11	Female	single	35	Oncology	MSc	7
P12	Male	Married	47	ICU	BSc	20

After data analysis using Colaizzi method, 110 initial concepts were extracted. These concepts emerged in 18 sub-themes and 6 main themes including: catastrophe, be in decline, be in progress, discrimination, work overload, and poor prognosis (Table 2).

**Table 2:** Themes, subthemes & codes

Themes	Subthemes	Codes
**Catastrophe**	Anxiety	feeling apprehensive feeling afraid
	Blame	feeling embarrassed feeling ashamed
	Burnout	Feeling exhausted
		tired nurse
	Precision supervision	to be under observation Feeling checked
**Be in decline**	Pressuring	Doing work using power Caring under pressure
	Disturbed care	Increasing error in nursing activity more complication for recommended patient
	Disrespected for the rules	Disturbance of the ward Disturbance in the nursing activity
	Displeasing Behavior	Punishment of the nurses Complaining about the nurse
	Impaired interaction	Conflict with the nurse Ordering to the nurse
**Be in progress**	Responsiveness	Handle the recommended patient with caution Being more responsible in recommended patient care
	Standard Care	More precise care Standard care
**Discrimination**	Violation of patient rights	Prioritize recommended patient care Out of the ordinary activity
	Disparity	Selecting nurses by authority Discrimination between patients
**work overload**	More work	Spending a lot of energy More care
	More time consuming	Time consuming recommended patient care Time consuming for responding to companions and family questions
	More follow-up	Frequent follow-up of recommended patient care Frequent refer from companions and family’s patients
**poor prognosis**	Death label	May God bless him/her The one who died
	Missed care	Missing patient Missed complications of the recommended patient

### Catastrophe

One of the main themes extracted from the interviews was catastrophe. In this study, “anxiety”, “blame”, “burnout”, and “Precision supervision” were the corresponding sub-themes of catastrophe.

Anxiety was one of the most important and repeated sub-themes in this study because the first reaction of the nurses in dealing with the recommended patient was the feeling of double stress and anxiety.

*“The recommended patient imposes more stress on the nurse and increases the psychological burden of the nurse. Even because of this stress and mental load, the treatment process of the recommended patient and other patients changes…” (P4).*

Also, nurses were afraid of taking care of these patients.

*“Taking care of the recommended patient gives me a feeling of dread. Really a feeling of dread. The recommended patient puts a lot of stress and nervousness on me.” (P1).*

#### Burnout

The recommended patient care had created a feeling of burnout and emotional exhaustion in nurses. Burnout is caused by being in unpredictable situations and enduring work and psychological pressure. Nurses felt helpless and forced to take care of recommended patients, and they felt exhausted due to the perception of coercive behaviors.

*“I feel helpless, it’s too bad that I know something, but a person who doesn’t know it forces me to do something wrong. When I see its complications on the patient, and the patient gets worse, I feel exhausted…” (P6).*

#### Blame

Nurses do their best to avoid mistakes in patient care, and feel ashamed and embarrassed if something goes wrong.

*“When the recommended patient has a problem while it was not our fault, we are worried and ashamed of that situation and we think that we were not able to carry out the colleague’s order properly…” (P4).*

#### Precision supervision

Nurses believe that taking care of a recommended patient is difficult and stressful because nurses are under direct supervision of ordering person and companions of the patient, and all their work is under supervision and they feel checked by the companions.

*“When a patient is ordered, the companions monitor us and give feedback to the superior or the ordering person. We feel that we are under strict observation and other non-experts are monitoring us, and this gives us a feeling of anxiety. (P7).*

### Be in decline

Another main theme from the interviews was the nurses’ experience of “be in decline”. In this research, “Pressuring”, “disturbed care”, “disrespected for the rule”, “displeasing behavior”, and “impaired interaction” were identified as the sub-themes of be in decline.

#### Pressuring

The participants believed that they are under a lot of psychological and work pressure in caring the recommended patients. They clearly stated that the exercise of power by the higher authority in the care of the recommended patient, ignoring the nurse, and work and behavioral restrictions cause a feeling of pressure in nurses.

*“Many times, officials who know that there are no empty beds and some patients are waiting for admission, call me and because they are superiors, I have to admit their recommended patient. In general, the work and behavior restrictions created by the patient are too many and it puts us under pressure…” (P1).*

#### Disturbed care

The nurses stated that due to the sensitivity of the recommended patients care, the companions and the ordering person usually interfere in the work of the nurses. These interferences disrupt the treatment process and ultimately harm the recommended patient.

*“They interfere with our work so much that we cannot do what we have always done properly. In think recommended patient syndrome often causes medical errors, because the companions feel that they are doing a favor to the patient, but they prevent the natural care process. They insist so much that the medical system sometimes loses its resistance in front of the expectations of these companions and the result is negative for the patient, because the wrong treatment ends up harming the patient…” (P6).*

Also, nurses considered the presence of recommended patients annoying and troublesome and declared their unwillingness to care for these patients.

*“I feel that the recommended patient is a trailing and troublesome phenomenon. I think its trouble is more than its benefits, because you have to answer the recommending person and many companions. Many times you have to explain to people who are not part of the patient’s main companion at all. Personally, I don’t like to care a recommended patient. I prefer to have many ordinary patients but not one recommended patient…” (P4).*

#### Disrespect for the rules

According to the participants, the presence of a recommended patient disturbs the order and regulations of the ward, and nurse’s work.

*“When a recommended patient is admitted, the ward becomes really disorganized. The ward looks messy and overworked. Because of them, we have to break a series of rules. It is forbidden to be accompanied in the intensive care unit (ICU), but not for those recommended! The companions of the patient are always there; they come and go whenever they want. The companions ignore the rules of the ward and even cause the patient’s work to be delayed. Working with the recommended patient disrupts the nurse’s work process…” (P9).*

#### Displeasing behavior

Nurses emphasized that they sometimes encounter inappropriate behaviors when caring for recommended patients. Complaining to the nurse by recommended patients or their companions, and reprimanding the nurse by the managers, caused the nurse to feel humiliated and threatened, this resulted in the appearance of the subtheme of displeasing behavior in this study.

*“For delay in a non-urgent work, the companions of the recommended patient sued me. The hospital authorities and even some of my colleagues condemned me. They said that we should give you another ward because of the big mistake you made. This matter lasted for several days. This caused some people to humiliate me, insult me, and create a very bad memory for me. I will never forget that scene, everyone gathered around me in the room and condemned and humiliated me…” (P11).*

#### Impaired interaction

In some communications between the nurse and the recommended patient and companions, nurses have felt humiliated. This humiliation has had negative and annoying effects on their morale and has caused a bad feeling. Inappropriate behavior with the nurse, giving orders to the nurse, conflict between the patient’s companions and the nurse, and inappropriate non-verbal communication have caused the nurse’s disrespect and impaired proper communication.

*“Many times, if a small mistake happens, the managers treat the nurse very harshly in order to show the patient’s companions or recommender that they prevent the mistakes. This humiliates the nurse and the nurse can no longer have a good relationship with the patient and companions…” (P6).*

### Be in progress

One of the main themes of this research was “be in progress”, which identified the positive aspects of nurses’ experiences in caring for recommended patients. “Standard care” and “responsiveness” were sub-themes of be in progress.

#### Responsiveness

Some nurses have been more cautious and responsible in taking care of recommended patients.

*“Nurses have a heavier duty to take care of the recommended patient and treat the patient carefully. As a nurse, we must be careful to do everything right. Although sometimes we do something unintentionally that still causes problems, but in general we have to be accountable to the recommended patient and our responsibility is greater…” (P4).*

#### Standard care

Although some participants stated that recommending a patient has no effect on nursing work, and care is provided without being recommended, but due to the sensitivity of the recommended patient, nurses tried to provide more accurate and standard care.

*“We try to do a more accurate and scientific care. For example, we put aside the works that we learned experimentally and try to implement those that are scientific so that a mistake does not happen. We do the work in a scientific and precise manner…” (P3).*

*“Recommended patients are somehow separated from other patients to receive care in a more special way and with a higher quality than ordinary patients. From the moment the patient arrives, everyone expects you to provide more special services, both the patients themselves and your superiors…” (P11).*

### Discrimination

Discrimination was recognized as another main theme. “Violation of patient rights” and “disparity” were the sub-themes of discrimination.

#### Violation of patient rights

All the nurses stated that the recommended patients expect their care to be prioritized and to be attended to as soon as possible without respecting the appointment. They believed that this would cause non-compliance with treatment justice and violation of the rights of other patients.

*“From the moment of arrival, recommended patients and superiors expect immediate services in the best way and in the fastest possible time. Recommended patients have expectations above the law. They think they should be a priority in all respects…” (P11).*

*“In general, recommended patients have high expectations. They want all their work to be done quickly and out of turn…” (P4).*

*“For the recommended patients, you have to spend more time for a series of tasks that are mostly unnecessary. As a result, you miss a series of necessary care for other patients, or you have to do it very quickly, or you don’t do it in the original time. You should always prioritize these patients, and this will violate the rights of other patients…” (P10).*

*“For example, the mayor had a patient in the ICU, he came with 4 other people, and I explained to him that you can’t stay for a few more minutes for the patient appointment. Although I respected him, he didn’t listen to me at all and they were there until the end of my shift. I did not have the power to take that person out of the ICU. Instead, I might say more forcefully to someone who isn’t recommended that it’s meeting time, you’re not allowed in the ward any longer. I really feel guilty and this is pure injustice. I say, when the recommended people don’t comply, why should others comply, and this is annoying…” (P8).*

#### Disparity

The participants stated that specific nurses with higher knowledge and more skill in clinical work are selected for the recommended patient, which will cause disparity and discrimination between patients and conflict between nurses.

*“They choose a special nurse for these patients. When they choose a special nurse for the recommended patient, they are actually discriminating between nurses. This choice means that some nurses are not reliable. This makes other nurses think that managers are not satisfied with their work and do not trust their work. The nurse thinks that I am a member of this system and they should treat me like everyone else, so they feel like a marginal person in the system, a person who is surplus…” (P3).*

### Work overload

Almost all nurses had experienced workload in caring for patients, which was identified in the three sub-themes of “more work”, “more time consuming”, and “more follow-up” due to excessive workload.

#### More work

Nurses stated that more care, spending more energy, and increasing work shifts for taking care of recommended patients imposes a heavy workload on them.

*“You must pay special attention to the recommended patients. Serve them in a special way and provide them with more care. In general, give more energy to perform care accurately and without mistakes, and this in itself imposes a lot of extra work on the nurse..” (P11).*

#### More time consuming

Most of the participants had to devote all their time to the recommended patient care in order to provide more care and spend more time on unnecessary tasks.

*“It is not fair to spend time on a patient who is not an emergency at all and does not need hospitalization, and it is coercive to hospitalize and continue hospitalization of this patient. This wastes my time and increases my workload because I have to provide much more time for patient care…” (P5).*

#### More follow-up

The addition of recommended patient follow-ups to routine care has created a heavy workload for nurses.

*“In the follow-up of giving medicine and the equipment that the patients must provide, we must fully explain to them that they must provide these equipment or drugs. For other patients, we may not be very persistent, but we have to be very persistent for recommended patients, and it is these follow-ups that cause an increase in workload…” (P7).*

### Poor prognosis

Poor prognosis was one of the main themes, which we separated into two sub-themes, “death label” and “missed care”. According to the nurses’ experiences of caring for recommended patients, they do not predict a good prognosis for them. In the nurses’ experiences in dealing with the recommended patient, there was a preconceived notion that the condition of the patients worsened and the end was inappropriate and sometimes the situation progressed to such an extent that a series of cares were unintentionally ignored.

#### Death label

Nurses’ experience of caring for recommended patients in some cases is that it is unlikely that their condition will improve.

*“When they say this is a recommended patient, we say, God bless him…” (P1).*

#### Missed care

Due to the sensitivities of the recommended patient, unwanted side effects are created that end up harming the patient, so that the nurses consider the care provided for the recommended patient as lost.

*“When the patient is recommended, we know that patient is missing. Really, when the term recommended patient comes, it is followed by the recommended patient syndrome. From our point of view, that patient is missed and lost…” (P1).*

## Discussion

This study was conducted aimed to explain the experiences of nurses with recommended patients. This study determined the outcomes of caring for recommended patients. One of the prominent themes in this study was Catastrophe. Anxiety and stress caused by the experience of nurses dealing with recommended patients was one of the important topics. In this regard, McIntosh also believes that caring for VIP patients is stressful for nurses [14]. This finding raises concerns about nurses’ burnout. The findings showed that nurses are afraid of taking care of these patients. In another studies, the fear and panic that occurs in care providers of VIP patients have been mentioned [24, 25]. Our findings showed that nurses often find themselves in sensitive and tense moments when dealing with recommended patients. Direct supervision by managers and companions makes care more difficult and creates fear in nurses. In taking care of recommended patients, nurses try not to make a mistake and if a mistake happens, they feel ashamed and embarrassed in front of the ordering person. Also, nurses experience additional work and psychological pressure, which leads to burnout in them. Sometimes there are relationships that are considered as an unwritten rule. For example, the relatives of the employees of the governorate, judiciary, doctors, etc., use extraordinary communication upon arrival at hospital and refer to the manager of the hospital, the head of the department, and even request special care for their patients. In addition, they do not follow routine rules for visiting hours, bedside hours, and even ordinary care [26]. These behaviors interfere with nurses’ work, and the stress caused by caring for these types of patients, exposes nurses to more pressure and more careful monitoring, which can make the presence of these patients unpleasant for nurses.

The second major theme was “be in decline”, included the negative consequences of caring for recommended patients. Nurses reported that they are under pressure from managers and companions to perform unnecessary care, which is consistent with the study by Allen-Dicker et al., [27]. Interference in the process of care and violation of rules leads to disruption of care and finally harming the patient, as in other studies it has been mentioned that the care of VIP patients is disrupted [3, 15]. In this study, nurses experienced violence from patients and their families in the form of a complaint to a higher authority, which was consistent with the study of Noor et al. [28]. The nurses emphasized that they are under pressure from the managers and the ordering person in taking care of recommended patients. This interference in caregiving leads to disruption of the nurse’s work, deviation from standard practices, increase in error, reduction in the quality of care, and ultimately harm to the patient. Other negative consequences include disorganization of the department, ignoring rules and regulations, inappropriate behavior such as complaining to the nurse, and inappropriate non-verbal communication that causes disrespect to the nurses and is associated with the reluctance of the nurses to provide care to these patients.

In contrast, “be in progress” shows the positive aspects of nurses’ experiences when caring for recommended patients. In caring for these patients, nurses try to provide special and excellent care. Also, the responsibility and accountability of nurses in dealing with these patients is more, which is in line with Atik’s study [29]. One of the positive consequences of caring for recommended patients is the accountability and responsibility of the nurses who tried to perform accurate and scientific care so that they would make fewer mistakes and not be blamed.

“Discrimination” was cited as a challenging aspect of nurses’ experiences with recommended patients. Nurses shared cases of discrimination between patients, faster work for recommended patients, selection of specific nurses for care, and concerns about providing equitable care, which were also mentioned in other studies [14, 29]. Recommended patients expect their care to be prioritized and their treatment and care to be without appointment, but this is contrary to justice treatment and violates the rights of other patients, and sometimes even disrupts the process of treatment and recovery of patients. On the other hand, ordering patients to managers creates a right for them, which leads to their objections and excesses. Also, other nurses believed that choosing a special nurse for a recommended patient causes discrimination between patients and conflict between nurses. These findings emphasize the importance of promoting equity in health care, and addressing implicit biases that can affect patient outcomes.

The “work overload” challenge emerged as another important issue in our study. Nurses performed unnecessary care of recommended patients in addition to the routine work of other patients. Unnecessary follow-ups, spending more time, and the time-consuming care of these patients impose more workload on nurses and contribute to nurses’ burnout. Khademi and colleagues have also pointed out the increase in the work pressure of nurses in caring for VIP patients [16]. Unnecessary follow-ups along with the urgent and real needs of the patient, the imbalance between the workload and energy of the nurses, and spending more time to care for these patients have increased the workload from the nurses’ point of view.

On the other hand, the experiences of nurses in dealing with such patients indicate an inappropriate outcome for these patients. It seems that focusing too much on these patients and performing additional procedures that are sometimes unnecessary exposes these patients to all kinds of harm and this issue may endanger the lives of patients.

### Limitations

Some of the limitations of this study included a relatively small sample size, limited to a specific geographic area, and nurses from limited wards. These factors may influence the generalizability of the findings. Future research can be done with larger samples and in other wards to confirm and expand the findings of this study.

## Conclusions

In this study, nurses understood their experiences of dealing with recommended patients in the form of catastrophe, be in decline, be in progress, discrimination, work overload and poor prognosis. Also, according to the results, the importance of the need to create culture and provide effective solutions should be considered. Therefore, in dealing with these patients, it is necessary to rely more on the existing laws in nursing, and the reputation and label of patients should not determine the way to care for them and for every patient regardless of its situation, care should be done to the fullest extent.

Understanding the experiences and challenges that nurses had in taking care of patients reminds us of the need for more empowerment and improvement of the working environment of nurses, including proper management of the working environment to facilitate caring for the all patients. Therefore, according to the consequences of these findings, educational programs and providing effective solutions should be considered. Therefore, it is suggested to conduct training courses in order to provide effective and quality care and increase the satisfaction of nurses, patients and their families.

## Data Availability

Datasets are available through the corresponding author upon reasonable request.

## Abbreviations

VIP: very important person
AACN: American Association of Critical Care Nurses
ICU: Intensive Care Unit
CCU: Cardiac Care Unit
BSc: Bachelor of Science
MSc: Master of Science
